# Playing and Listening to Tailor-Made Notched Music: Cortical Plasticity Induced by Unimodal and Multimodal Training in Tinnitus Patients

**DOI:** 10.1155/2014/516163

**Published:** 2014-05-08

**Authors:** Janna Pape, Evangelos Paraskevopoulos, Maximilian Bruchmann, Andreas Wollbrink, Claudia Rudack, Christo Pantev

**Affiliations:** ^1^Institute for Biomagnetism and Biosignalanalysis, University of Münster, Malmedyweg 15, 48149 Münster, Germany; ^2^ENT Department, University Clinic Münster, University of Münster, Kardinal-von-Galen Ring 10, 48149 Münster, Germany

## Abstract

*Background*. The generation and maintenance of tinnitus are assumed to be based on maladaptive functional cortical reorganization. Listening to modified music, which contains no energy in the range of the individual tinnitus frequency, can inhibit the corresponding neuronal activity in the auditory cortex. Music making has been shown to be a powerful stimulator for brain plasticity, inducing changes in multiple sensory systems. Using magnetoencephalographic (MEG) and behavioral measurements we evaluated the cortical plasticity effects of two months of (a) *active* listening to (unisensory) versus (b) learning to play (multisensory) tailor-made notched music in nonmusician tinnitus patients. Taking into account the fact that uni- and multisensory trainings induce different patterns of cortical plasticity we hypothesized that these two protocols will have different affects. *Results*. Only the *active* listening (unisensory) group showed significant reduction of tinnitus related activity of the middle temporal cortex and an increase in the activity of a tinnitus-coping related posterior parietal area. *Conclusions*. These findings indicate that *active* listening to tailor-made notched music induces greater neuroplastic changes in the maladaptively reorganized cortical network of tinnitus patients while additional integration of other sensory modalities during training reduces these neuroplastic effects.

## 1. Introduction

Subjective tinnitus is an auditory perception in the absence of physical sources [1, 2]. While transient tinnitus lasts only a couple of seconds to a few hours, chronic tinnitus is an ongoing conscious perception of a sound for more than three months with low incidence of spontaneous remissions. Around 5–15% of the people in western countries suffer from chronic tinnitus affecting their quality of life, that is, sleep disturbance, work impairment, and psychiatric distress [3]. An investigation of the individual characteristics of tinnitus in 528 tinnitus patients by [4] showed that about 65% of the tinnitus patients suffer from tonal tinnitus.

Tinnitus perception is often associated with aging and hearing loss; it arises in auditory cortex, and the generation and maintenance have been associated with maladaptive reorganization of the auditory cortex [5]. Following certain tinnitus trigger events such as noise or stress, the central auditory pathway reorganizes itself, exhibiting excitatory-inhibitory network dysbalances and permitting increased spontaneous firing rates, burst firing, and neuronal hypersynchrony [6, 7]. Physiological studies in mice suggest that the most probable underlying mechanism of this reorganization consists of the loss of inhibitory drive to neurons, elicited by changes in glycinergic [8] and GABAergic [9] systems. Upregulations of glutamatergic and cholinergic systems may be involved as well [10, 11]. These changes affect several levels of the auditory pathway along with nonauditory centers comprising a network that includes posterior parietal, frontal, somatosensory, and limbic regions [12–14]. Importantly, the auditory cortex activity corresponding to the tinnitus frequency has been consistently shown to be enhanced and related to perceived tinnitus loudness [5, 15, 16].

Causal tinnitus therapies are not yet widely available, but recent neurophysiological studies indicate that modality appropriated training can reverse maladaptive cortical reorganization [17–19]. Recent MEG studies [20–23] indicate that short-term and long-term listening to spectrally “notched” music (tailor-made notch-music, TMNM) containing no energy in the frequency range at and around the individual tinnitus frequency can considerably reduce the tinnitus-related neuronal activity of primary and nonprimary auditory cortical structures and alleviate tinnitus perception through lateral inhibition. In the abovementioned studies that introduced this approach, TMNM did not include active engagement with the music. Instead, the task of the tinnitus patients during the training was merely to listen to their favorite music. Nevertheless, attention plays an important but still unclear role in tinnitus perception [24–27] and the corresponding change in cortical plasticity [28].

Music playing is a highly complex task. It involves almost all sensory systems as well as the motor system and requires high amount of precision and accuracy with regard to the coordination and integration of the different sensory systems [29]. Therefore, extensive music training induces plastic changes in the human brain on both functional [30] and structural [31] levels. Recent studies indicate that even short-term (1-2 weeks), laboratory controlled music training can induce cortical plasticity [32] while its multisensory component plays a crucial role increasing the resulting plasticity effects [33, 34].

Therefore the goal of this MEG and behavioral study was to compare the neuroplastic effects of uni- and multimodal music trainings by manipulating the focus of attention. Two groups of nonmusicians suffering from chronic, tonal tinnitus were investigated. In the multimodal group, subjects were trained to play simple melodies on a tablet computer accompanying preset music songs. Thus, their attention was almost equally divided to all sensory systems involved in the task: visual, sensory-motor, and auditory. In contrast, in the unimodal group, high degree of attentional demands was introduced by asking the subjects to detect small auditory variations in repeated runs of the songs. Hence, the* focus* of attention was either solely in the listening (auditory modality, unimodal group) or divided to the somatosensory, visual, and auditory modality (multimodal group). The music of both groups was filtered in real time over headphones with a notch filter surrounding the individual tinnitus frequency. Results were evaluated using neurophysiological and behavioral pre-, during, and posttraining measurements.

## 2. Materials and Methods

### 2.1. Subjects

Twenty-six tinnitus patients were recruited by advertisement in local newspapers. Informed consent was obtained by procedures consistent with the principles of the Declaration of Helsinki and approved by the Ethics Commission of the Medical Faculty, University of Münster. In order to participate in the study subjects had to fulfill the following criteria with respect to their tinnitus: (i) chronic (≥3 months), single stable (no pitch fluctuation) tonal tinnitus perception (beep- or whistle-like), (ii) tinnitus frequency ≤8.5 kHz (to ensure unrestricted sound stimulation), (iii) age ≤ 65 years, (iv) no severe hearing impairment (hearing loss ≤ 55 dB HL in the frequency range from 0.125 to 8.5 kHz), (v) no psychological or neurological diseases, (vi) no current alcohol or drug abuse, (vii) no parallel tinnitus treatment, and (viii) no training in playing an instrument. Subjects were pseudorandomly assigned to the unimodal (listening) or multimodal (playing) group. For homogeneity matching the following criteria were also considered: (i) tinnitus pitch, (ii) time since tinnitus onset, (iii) age, (iv) hearing loss, (v) subjective tinnitus loudness, (vi) Tinnitus Questionnaire (TF) [35], and (viii) Symptom Check List SCL 90R total score [36].

Over the course of the study, three subjects dropped out due to lack of time for training; one subject had the impression of possible tinnitus worsening; thus the dropout rate per group was playing group (2/13) and listening group (2/13). Three subjects were not included in the MEG analyses due to extensive hearing loss that did not allow sufficiently loud auditory stimulation: playing group (1/11) and listening group (2/11). Finally, 19 subjects completed the 3-month study (2 months of music training and 1 month followup) and were included in the MEG-data evaluation: playing group *n* = 10 and listening group *n* = 9.

On average (mean ± SD), the two groups did not differ significantly in age (46.3 ± 11.66 years, range 23–64 years, *P* = 0.78) and average hearing loss (19.93 dB SL ± 12.41; range 5–55 dB SL, *P* = 0.66) or the tinnitus characteristics (i) duration (35.42 ± 14.20 years; range 14.49–56.15 years, *P* = 0.81), (ii) frequency (5.954 kHz ± 2.136; range 1–8.5 kHz, *P* = 0.86), (iii) loudness estimate of tinnitus (55.64 ± 26.23; range 16–99; scale 0–100, *P* = 0.13), or tinnitus-related distress in the Iowa tinnitus handicap questionnaire total score (27.78 ± 15.77; range 5.93–57.78, *P* = 0.69) and in the SCL-90-R (0.32 ± 0.25; range 0.03–0.84, *P* = 0.8). The abovementioned characteristics of the sample are summarized in Table 1. Most of the subjects reported bilateral tinnitus (bilateral: *n* = 17; left lateralization: *n* = 2; right lateralization: *n* = 3).

### 2.2. Design

**Figure 1:**
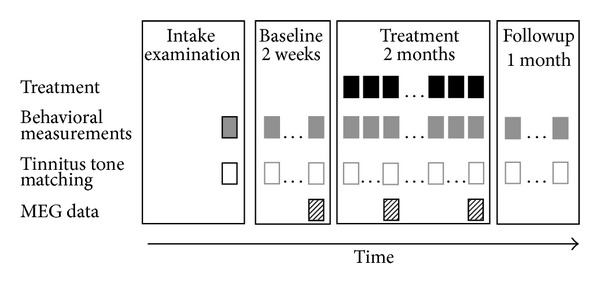
Illustration of the design. The red squares indicate the training sessions, the blue ones the behavioral measurements, the yellow ones the tinnitus tone matching measurements, and the green ones the MEG measurements.

The design of the study comprised three parts. The first part (baseline) lasted 2 weeks and included 2 weekly measurements of the subjective tinnitus characteristics. The second part lasted 2 months and included, for both groups, one hour of daily training (described in detail below), 8 weekly measurements of the subjective tinnitus characteristics, and 3 MEG recording sessions: one prior to the training, one after one month of training, and one after completion of the training. The third part lasted one month and included 4 weekly measurements of the subjective tinnitus characteristics as a followup. An illustration of the design is shown in Figure 1.

During the performance of the experiment a tablet computer with a touch screen (iPad-II, Apple Inc) was provided to each patient including a music application (ThumbJam https://itunes.apple.com/us/app/thumbjam/id338977566?mt=8) that served as the basis for the musical training of the two groups. The main user interface of this music application resembled a simplified piano keyboard. An in-house application regarding tinnitus frequency likeness rating (TPLR) was also installed. Moreover, a set of headphones was provided (Sennheiser, PX360) which was modified by the company enabling us to program the filter coefficients of the (active) noise cancellation, based on the individual tinnitus frequency, in a way that an online notch filtering could be performed while listening to music.

### 2.3. Training

#### 2.3.1. Multimodal (Playing) Group

The subjects of the playing group were instructed in detail how to use the provided tablet computer and the music application. Thirty different songs, each in three various tempi, had to be melodically accompanied by pressing the correct position on the touch screen of the tablet, on the basis of a self-created music book suitable for nonmusicians. The song difficulty increased over time and new learned songs had to be repeated the next day. Subjects could choose their favorite finger technique using either all fingers (as written in the music book) or only the thumbs of both hands. Training duration was one hour per day and the training sessions were recorded weekly. While playing, the subjects were listening to the melody they played along with the preset backing track. All music spectra were notched in real time via the provided headphones.

#### 2.3.2. Unimodal (Listening) Group

The subjects of the listening group used the provided tablet for listening to the same 30 songs as the playing group. In order to increase the amount of attention needed, the subjects had to fulfill an auditory task while listening to the music. All songs were played in two runs. The first run was played in the correct way as it was written in the music book. A second run directly followed the first one providing the same song. The second run was either identical or contained up to six variations that had to be detected by the patient. After each pair of songs the identified number of variations had to be filled in a form. Each session lasted one hour comprising all 30 songs in a randomized order. In the course of the study the difficulty of the variations increased (from dissonant to consonant variations) and new variations were repeated the next day. As in the playing group, all music spectra were notched in real time via the provided headphones.

### 2.4. Intake Examination

All subjects were recruited by the tinnitus team and completed a structured interview that collected information on the nature and the personal history of their tinnitus. Audiological measurements included an otoscopic examination, securing that the subjects do not suffer from objective tinnitus. Then, measurements of the hearing threshold with a high-frequency audiometer (0.125 to 16 kHz) and determination of the tinnitus frequency following a structured audiological protocol, using a frequency resolution of 1/24 octave, were performed. Further, the subjects had to assess their tinnitus loudness, distress, awareness, and handicap over the last three days by visual analogue scales. An assessment of tinnitus distress followed with a battery of tests that are described in the subsequent section.

### 2.5. Measurement of Subjective Tinnitus Characteristics

#### 2.5.1. Frequency

Two procedures were applied in order to determine the tinnitus frequency. (i) Seven “tinnitus frequency candidates” were collected by professional audiologists at the ENT department following the same procedure as described by H. Okamoto at al., 2010. Specifically, tinnitus pitch and loudness were matched ipsilaterally to the frequency and loudness of a pure tone starting from seven different anchor frequencies (1, 12.5, 2, 10, 4, 8, and 6 kHz). Next, two of the previously determined tinnitus frequency candidates were directly compared in a two-alternative forced-choice (2AFC) procedure and the winner of each comparison was tested against the lowest remaining candidate frequency until the winner tinnitus frequency was found. In an octave confusion test the loudness-matched harmonics of the winner tinnitus frequency were again directly compared in a 2AFC procedure. (ii) The subjects were asked to assess their tinnitus frequency at home on the seven following days using a tinnitus pitch likeness rating (TPLR) application on the provided tablet computer. In this process 37 loudness-matched test tones (sinusoidal tones, two minutes duration, two seconds fadein, and one second fadeout) in frequency steps of 1/12 octave from 2 kHz to 16 kHz (three octaves) had to be rated according to the tinnitus likeness on a scale from 0 to 10 points. The test tones were presented in a randomized order each day. After seven days five tones with the highest ratings including the winner tinnitus frequency of (i) were directly compared in a 2AFC procedure. An octave confusion test was applied on the winner tinnitus-frequency of the TPLR. Afterwards, the audiometric pitch matching described in (i) was repeated: the winner tinnitus frequency of the audiometric approach was compared with the winner tinnitus frequency of the TPLR. This last 2AFC determined the tinnitus frequency for the following TMNM treatment. Over the course of the study, additional TPLR measurements were obtained regularly one time per week.

#### 2.5.2. Tinnitus Related Distress

Tinnitus related distress was assessed with the German translations of (i) Tinnitus Handicap Questionnaire (THQ), (ii) the tinnitus handicap inventory (THI), and (iii) the Tinnitus Questionnaire (TQ). Hyperacusis was assessed with the Geräuschüberempfindlichkeits-Fragebogen (GÜF) [37] and subjective impairment was valued by the SCL-90R. Psychic constitution is estimated by the ADS-L and the state-trait-anxiety-inventory (STAI) [38]. All subjects were asked to estimate their duration of music listening per day, their fun, and relaxation while listening. All questionnaires were fulfilled at the beginning of the training and after each of the following three months.

#### 2.5.3. Tinnitus Characteristics and Evaluation of TMNM Treatment

Tinnitus loudness, awareness, distress, and handicap were measured twice a week on a continuous visual analog scale performed on the provided tablet computer (scale poles: 0 (= tinnitus gone) versus 100 (= personal tinnitus loudness maximum experienced so far)). A baseline period of two weeks before the music training was surveyed. Subjects were also asked to estimate their progress in the music training, the difficulty of the training, their fun, and motivation to continue.

### 2.6. MEG Measurement Stimuli

Two different sound stimuli were prepared and delivered randomly to either the left or the right ear during the MEG measurement via 60 cm long silicon tubes. The frequency of one stimulus corresponded to the tinnitus frequency; the control stimulus had a frequency of 500 Hz. The loudness of the control stimulus was 20 dB above sensation level that was determined with an accuracy of at least 5 dB at the beginning of each MEG session for each ear. The tinnitus frequency was matched in loudness to the control stimulus before the baseline measurement and kept identical across all course measurements. The stimuli had duration of one second and a random onset asynchrony between two and three seconds. Four runs were presented lasting approximately 14.5 min each, with short breaks in between. The total amount of stimuli for each category was 500.

### 2.7. MEG Recordings

Evoked magnetic fields were measured with a 275-channel whole-head system (OMEGA, CTF Systems Inc, Port Coquitlam, Canada) in a magnetically shielded and acoustically silent room. MEG data were acquired continuously during each run with a sampling rate of 600 Hz. Subjects were seated upright, and their head position was comfortably stabilized with cotton pads inside the MEG dewar. During the four measuring runs the subjects watched a soundless video of their own choice projected onto the back of a semitransparent screen positioned 90 cm in front of the subjects' nasion. Between runs two and three and after the last run the subjects had to rate their tinnitus loudness on a visual analogue scale.

### 2.8. Data Analysis

Brain Electrical Source Analysis software (BESA research, version 5.3.7, Megis Software, Heidelberg, Germany) was used for the processing of the MEG data. The recorded data were separated into epochs of 700 ms including a prestimulus interval of 200 ms. The epochs were baseline corrected using the interval from −100 to 0 ms. Epochs with amplitudes larger than 2.5 pT were considered as artifacts and were excluded from the averaging procedure. Data were filtered off-line with a low-pass filter of 30 Hz and a high-pass filter of 1 Hz. Current density reconstructions (CDR) were calculated on the brain responses of each subject for each stimulus category (tinnitus tone and control tone) and each one of the four runs using the LORETA method [39]. LORETA directly computes a current distribution throughout the full brain volume instead of a limited number of dipolar point sources or a distribution restricted on the surface of the cortex. This method has been used successfully for the mapping of auditory evoked brain responses [34, 40] and has the advantage of not needing an a priori definition of the number of activated brain sources. A time window of 50 ms was used for the CDR (70–120 ms after stimulus onset). The chosen time window contains the typical latency of the N1 component ranging from 70–120 ms and includes the rising slope and the peak of the grand average global field power (GFP) of the responses within this time range. Each individual's mean CDR image over the selected time-window for each one of the 4 runs was calculated and projected onto a standard MRI template based on the Montreal Neurological Institute (MNI) template. The images were smoothed and their intensities normalized by convolving an isotropic Gaussian kernel with 7 mm full width half-maximum (FWHM) through BESA's smoothing utility. The smoothed images of each run were then averaged in order to achieve a sufficient signal to noise ratio, producing thus one image for each condition (control and tinnitus) and each time-point (before training, after one month of training, and after training) of each subject.

The software packages Statistical Parametric Mapping 8 (SPM8, http://www.fil.ion.ucl.ac.uk/spm) and GLM-Flex (http://nmr.mgh.harvard.edu/harvardagingbrain/People/AaronSchultz/GLM_Flex.html) were used for the statistical analysis of the CDRs. Specifically, using GLM-Flex a 2 × 2 × 3 mixed model ANOVA was designed with subjects factor group (playing and listening) and within subjects factors frequency (tinnitus and control) and time point (before training, after one month of training, and after training). Results were constrained in gray matter using a mask, thereby keeping the search volume small and in physiologically reasonable areas. A permutation method for peak cluster level error correction (AlphaSim) at *P* = 0.05 was applied for this whole head analysis, as implemented in REST software (Song et al., 2011), by taking into account the significance of the peak voxel (threshold *P* < 0.001 uncorrected) along with the cluster size (threshold size > 513 voxels), thereby controlling for multiple comparisons. The smoothness factor used for AlphaSim estimation was calculated from the residual image of the three-way interaction effect.

## 3. Results

### 3.1. Behavioral Results

The four items measuring the tinnitus severity via visual analogue scales (tinnitus loudness, awareness, distress, and handicap) were highly intercorrelated (Cronbach's *α* = 0.968; averaged over the 14 weekly measurements). Consequently, we chose to average the four items to obtain a single measure of perceived tinnitus* severity*.

The first two time points defined the baseline. Severity values at baseline appeared to differ between the listening and the playing group (*M*_listen_ = 43.14, SEM_listen_ = 7.41; *M*_play_ = 56.49, SEM_play_ = 6.98). Although a *t*-test showed that this difference was not significant, *t*(20) = 1.233, *P* = 0.232, we chose to analyze the development of tinnitus severity by means of an analysis of covariance (ANCOVA) to control for possible baseline effects due to accidentally imbalanced sampling. For each of the 12 postbaseline measurement points we calculated the change-from-baseline severity and performed a 2 (hroup) × 12 (time point) ANCOVA, with* baseline severity* as a covariate.

**Figure 2:**
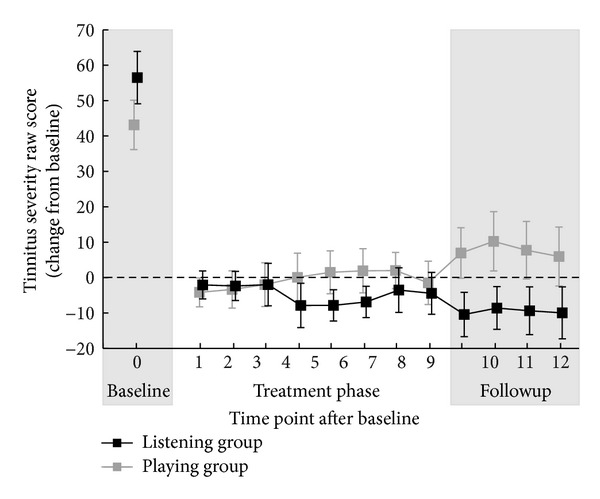
Behavioral data. Subjective tinnitus severity score (scale ranged from 0 to 100) for the playing group (gray) and listening group (black). Time point 0 corresponds to the baseline. For time points 1 to 12 the figure depicts the change from baseline in tinnitus severity. Error bars show ± 1 SEM.

For the factor time point we observed a violation of the sphericity assumption (Mauchly's *W* = 0.00001, *P* < 0.001) and will thus report Greenhouse-Geisser corrected *P* value where necessary. Only the interaction time point × group was marginally significant, *F*(11, 209) = 1.784, *P* = 0.058, indicating that the development of severity over time could be predicted from baseline severity. No other effects were significant (all *P* > 0.149).  Figure 2 illustrates the development of severity change from baseline over time. No significant effects were seen in the other behavioral measurements used (THQ, THI, TQ, SCL-90R, ADS-L, and STAI).

### 3.2. MEG Results

Our main hypothesis states that the two training types should develop different effects between the groups over the course of the training, but exclusively for the tinnitus frequency, not for the control frequency. The relevant statistic test is therefore a three-way interaction of group, frequency, and time point. This analysis is run first and is henceforth used as a localizer; that is, all further analyses that are performed to resolve the three-way interaction will be restricted to cortical regions where the three-way interaction was found to be significant.

**Figure 3:**
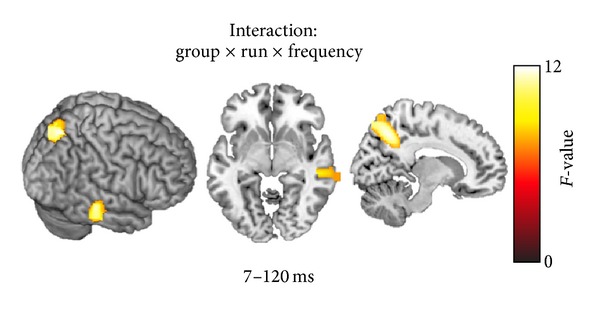
Statistical parametric maps of the group × frequency × time point interaction. The tailor made notched music training affected in a significantly different way the two groups and the two frequencies in two areas: right middle temporal cortex, (peak coordinates: *x* = 56, *y* = −28, and *z* = −8; *F*(2, 34) = 11.492; cluster size = 766 voxels; *P* < 0.05 AlphaSim corrected) and right posterior parietal cortex (peak coordinates: *x* = 12, *y* = −66, and *z* = 48; *F*(2, 34) = 11.816; cluster size = 1208 voxels; *P* < 0.05 AlphaSim corrected).

The statistical comparison of the MEG results indicated that TMNM treatment affected differently the cortical responses of the two groups and two frequencies. Specifically, the three-way interaction of the mixed model ANOVA (group × frequency × time point) yielded two significant clusters: one at the right middle temporal cortex (peak coordinates: *x* = 56, *y* = −28, *z* = −8; *F*(2, 34) = 11.492; cluster size = 766 voxels; *P* < 0.05 AlphaSim corrected) and one in Brodmann area 7 at the posterior parietal cortex (peak coordinates: *x* = 12, *y* = −66, *z* = 48; *F*(2, 34) = 11.816; cluster size = 1208 voxels; *P* < 0.05 AlphaSim corrected). The statistical parametric map of this analysis is presented in Figure 3.

In order to investigate the origin of this result a mask was constructed that included only the two clusters that were found to have significant effects in the abovementioned three-way interaction (i.e., right temporal cortex and posterior parietal cortex). This mask was then used as region of interest (ROI) for the post hoc analyses of the two-way interactions of frequency × time point for each group. The two-way interaction in the analysis of the playing group revealed no significant activation differences, even when the threshold was lowered at an uncorrected *P* < 0.01 level. On the contrary the two-way interaction of the listening group showed significant activation differences in both cortical areas (right temporal cortex and posterior parietal cortex) in the AlphaSim corrected *P* < 0.05 threshold level, thereby indicating that the three-way interaction originated from a two-way interaction that was more pronounced in the listening than in the playing group (peak coordinates for the right temporal activation: *x* = 43, *y* = −29, *z* = −13; *F*(2, 16) = 12.4059; cluster size = 569 voxels; *P* < 0.05 AlphaSim corrected; peak coordinates for the posterior parietal activation: *x* = 7, *y* = −59, *z* = −42; *F*(2, 16) = 10.3954; cluster size = 2097 voxels; *P* < 0.05 AlphaSim corrected). A two-way ANOVA of group × time point only for the tinnitus frequency was then calculated that revealed a significant activation difference between the two groups and the 3 time points (peak coordinates for the right temporal activation: *x* = 43, *y* = −29, *z* = −13; *F*(2, 34) = 7.5943; cluster size = 364 voxels; *P* < 0.05 AlphaSim corrected; peak coordinates for the posterior parietal activation: *x* = 7, *y* = −59, *z* = −42; *F*(2, 34) = 6.82; cluster size = 146 voxels; *P* < 0.05 AlphaSim corrected).

**Figure 4:**
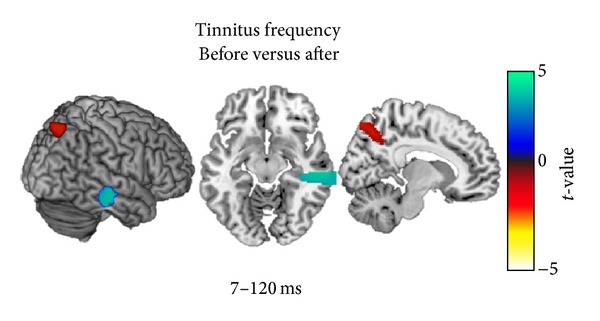
Statistical parametric maps of the post hoc paired samples *t*-tests ROI comparing the pre- and posttraining MEG results of the listening (unimodal) group with regard to the tinnitus frequency. Tailor made notched music training induced a decrease in the activity of the right temporal cortex (peak coordinates: *x* = 68, *y* = −22, and *z* = −8; *t*(16) = 3.62; cluster size = 472 voxels; *P* < 0.05 AlphaSim corrected) and an increase in the activity of the posterior parietal cortex (peak coordinates: *x* = 12, *y* = −64, and *z* = 52; *t*(16) = −3.64; cluster size = 155 voxels; *P* < 0.05 AlphaSim corrected).

**Figure 5:**
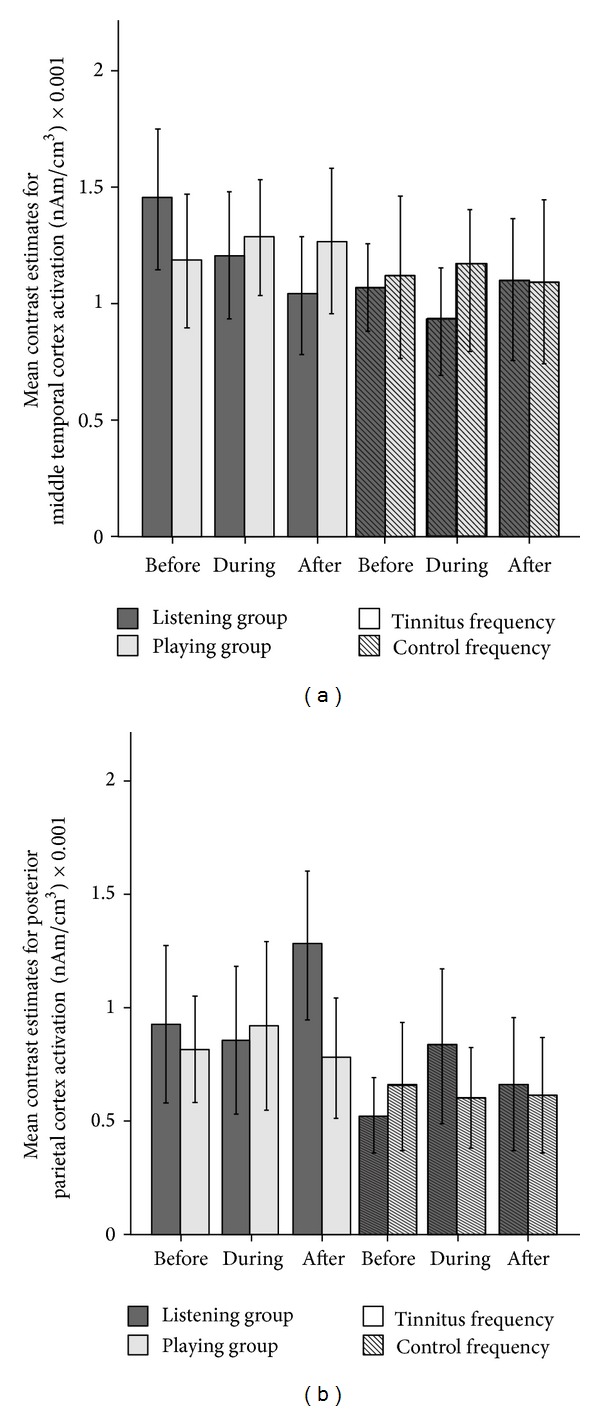
Mean contrast estimates for (a) the middle temporal cortex and (b) posterior parietal cortex before, during, and after the treatment. The solid dark grey bars indicate the contrast estimates of the listening group while the solid light grey indicates the activations of the playing group for the tinnitus frequency. The dark grey bars marked with lines indicate the contrast estimates of the listening group for the control frequency. The light grey bars marked with lines indicate the contrast estimates of the playing group for the control frequency. The treatment caused a gradual decrease of the activation of the middle temporal cortex and an increase in the activation of the posterior parietal cortex in the tinnitus frequency of the listening group. Error bars: 2 × standard error of mean.

Subsequently, using the same mask and threshold two post hoc one-way ANOVAs (one for each frequency) with factor time point within the listening group were calculated. The analyses showed activation differences in the regions of interest defined by the three-way ANOVA only in the tinnitus frequency (peak coordinates for the right temporal activation: *x* = 68, *y* = −20, *z* = −8; *F*(1, 16) = 13.50; cluster size = 144 voxels; *P* < 0.05 AlphaSim corrected; peak coordinates for the posterior parietal activation: *x* = 14, *y* = −66, *z* = 56; *F*(1, 16) = 13.27; cluster size = 187 voxels; *P* < 0.05 AlphaSim corrected), while no activation difference was found for the control frequency, thereby indicating that the TMNM treatment affected the responses to the tinnitus frequency but not to the control frequency. To identify the direction of this result a paired sample *t*-test of before to after the tinnitus frequency was calculated for the listening group (again using the same threshold). Thereby it was revealed that the response of the right temporal cortex for the tinnitus pitch for the listening group decreased during the course of the treatment (peak coordinates: *x* = 68, *y* = −22, *z* = −8; *t*(16) = 3.62; cluster size = 472 voxels; *P* < 0.05 AlphaSim corrected), while the response of the posterior parietal cortex increased (peak coordinates: *x* = 12, *y* = −64, *z* = 52; *t*(16) = −3.64; cluster size = 155 voxels; *P* < 0.05 AlphaSim corrected). The statistical parametric maps of this analysis are presented in Figure 4 and the mean contrast estimates of the 2 regions for the 3 time points (i.e., middle temporal cortex and posterior parietal cortex) are presented in Figure 5.

## 4. Discussion

In this study we compared the cortical plasticity effects of multimodal and unimodal notched music treatment in tinnitus patients by means of MEG and behavioral measurements over a time period of three months (two months of TMNM treatment for one hour per day and one month followup). Results indicate a decrease in the cortical activity corresponding to the tinnitus frequency for the unimodal training group, while no significant effect was present in the multimodal group. Importantly, the MEG results reveal, for the first time according to our knowledge, that unimodal TMNM treatment induces favorable plastic cortical changes not only in the temporal cortex, but also in a posterior parietal region, which constitutes another node of the cortical network that underlies the generation and/or maintenance of the tinnitus perception [41–43].

The present study employed an active engagement with music in both the uni- and multisensory groups: one group detected variations in the preset music pieces and the other one melodically accompanied preset backing tracks. At the same time, the acoustic input was filtered in real time with a tailor made notch filter targeting the individual tinnitus frequency via the supplied special type of headphones. In the unisensory group this process caused a decrease in the temporal cortex responsiveness to the tinnitus frequency, while it did not affect the response to the control frequency. As shown in previous studies [18, 20, 22], listening to pleasurable tailor-made notched music can reduce tinnitus perception and reduce the evoked activity in temporal cortex areas corresponding to the tinnitus frequency. This kind of individually modified music introduces a functional deafferentation of auditory neurons corresponding to the eliminated frequency while the surrounding neurons, which are still excited by the nonfiltered acoustic input presumably suppressing the tinnitus generating neurons via lateral inhibition [21, 44, 45]. Thus, the deprivation from auditory input in the frequency range of the tinnitus seems to induce long-term depression in auditory neurons corresponding to the tinnitus frequency via synaptic and/or cellular mechanisms [46, 47]. This process seems to affect mainly the right temporal cortex due to an increased predisposition of right auditory cortical neurons to synchronize their activity following deafferentation leading to tinnitus [48].

The specificity of the right auditory cortex in processing spectral information [49, 50] in contrast to the left one, that is, specified in the processing of temporal auditory information [51] along with the fact that tinnitus distress, is highly related to the activity of right temporal areas which [52] may be the reason for the right lateralized effect of the applied treatment. The neuroplastic effect of the treatment is located in the MTG. This area is correlated with auditory awareness of pitch [53] contributing, thus, to the perception that the tinnitus sound is externally located [54]. A recent voxel based morphometry study by Boyen et al. [55] revealed that tinnitus is associated with higher grey matter volume in MTG, while a meta-analysis of tinnitus related PET studies [56] indicated increased activation in MTG in tinnitus patients. Hence, the treatment effect of decreased activity in MTG may indicate a functional reorganization of the temporal network that subserves tinnitus [54].

Additionally, the training caused an increase of the activity of the posterior parietal cortex as a response to the tinnitus frequency in the unisensory group. For this region positive correlation between glucose metabolism and tinnitus was reported in a recent PET study [42], while its activation in tinnitus patients has been also shown in previous PET study [57]. Importantly, an increase of the activity in the posterior parietal cortex (precuneus) has been found to positively correlate with less tinnitus distress in recent EEG studies [14, 58, 59], indicating that it may constitute part of a tinnitus coping network. This interpretation seems plausible as this region has also been correlated with selective attention in the auditory modality [60]. Within this framework, the fact that TMNM treatment causes an increase in responsiveness of the posterior parietal cortex becomes increasingly important.

In a series of studies music making has been shown to be one of the most powerful stimulators for brain plasticity, inducing changes in multiple sensory systems [32, 61]. Three recent training studies using MEG indicated that music training based on a multisensory protocol that utilizes the auditory, visual, and motor modalities enhances the plastic changes induced by musical training in healthy adult nonmusicians [33, 34, 62]. The reward and enjoyment of playing music compared to merely listening to it seems to cause an increase in dopamine release that can enhance cortical reorganization [63]. Nevertheless, in the present study only the unisensory (listening) but not the multisensory (playing) group revealed a significant effect. Thereby we assume that mechanisms reversing the changes in the auditory system, which have been already reorganized in a maladaptive manner generating tinnitus perception, are not the same as the mechanisms that drive the cortical plasticity induced by music training in healthy adults [32]. Instead, the amount of attention dedicated to the auditory input seems to be even more crucial. Attention strengthens not only the excitatory neural connections but also the inhibitory networks, thereby driving also the effectiveness of tailor-made notched music in the auditory system [60]. In the training protocol of the present study the listening group concentrated on the auditory input solely, while the playing group divided its attention to the different modalities, that is, the somatosensory (pressing a button with the right finger in time on the tablet), visual (reading the music book), and auditory system (listening to music).

The behavioral responses on the visual analog scales with regard to tinnitus severity do not reflect the changes observed in MEG. This null-finding is in contrast to former studies using TMNMT [20, 22, 23]. This result can be attributed to a combination of small sample size (*n* = 9 for the listening group and *n* = 10 for the playing group) and the great interindividual variance included in the data (cf.  Figure 2). Moreover, the training lasted two months which is a considerably smaller time period compared to other studies [20] and therefore slight differences in tinnitus perception may have not been detected with the questionnaires [64].

## 5. Conclusion

Listening attentively to individually filtered music over a time period of two months, for one hour per day, led to plastic cortical changes in a network of sources that subserve the generation and/or maintenance of tinnitus, as revealed by MEG measurements (a decrease of auditory evoked activity in the right temporal cortex and an increase of activity in the posterior parietal cortex). The present study also indicates that unimodal tailor-made notched music training induces greater neuroplastic changes than multimodal training in nonmusician tinnitus patients. Thereby we assume that the mechanisms reversing the maladaptively reorganized auditory system that generates tinnitus perception are different from the mechanisms driving the cortical plasticity induced by music training in healthy brains. Thus, a training protocol based on attentive listening to tailor-made notched music can reverse the maladaptive reorganization of the cortical network that generates and supports tinnitus perception.

## Figures and Tables

**Table 1:** Characteristics of the sample of the study.

	Group	Mean	SD	SEM
Age (y)	Playing	45.55	12.00	3.79
	Listening	46.97	11.86	3.43

Hearing loss (dB)	Playing	18.60	11.25	3.56
	Listening	21.04	13.69	3.95

Duration (y)	Playing	34.61	11.89	3.76
	Listening	36.09	16.38	4.73

Pitch (hz)	Playing	5865.00	2094.18	662.24
	Listening	6029.17	2261.18	652.75

Subjective loudness	Playing	46.41	24.08	7.62
	Listening	63.33	26.40	7.62

THI	Playing	26.20	20.01	6.33
	Listening	29.67	19.78	5.71

SCL90_R	Playing	0.34	0.26	0.08
	Listening	0.31	0.26	0.07

## List of Abbreviations

MEG: Magnetoencephalography
TMNMT: Tailor-made notch-music training
ANOVA: Analysis of variance
AlphaSim: It is used in multiple regression analysis to find the minimum cluster size for certain alpha
TPLR: Tinnitus frequency likeness rating
2AFC: Two-alternative forced-choice
THQ: Tinnitus Handicap Questionnaire
THI: Tinnitus handicap inventory
TQ: Tinnitus Questionnaire
GÜF: Geräuschüberempfindlichkeits-Fragebogen
STAI: State-trait-anxiety-inventory
ADS-L: Allgemeine depressionskala lange version
BESA: Brain Electrical Source Analysis Software
CDR: Current density reconstructions
LORETA: Low resolution brain electromagnetic tomography
GFP: Global field power
MRI: Magnetic resonance imaging
MNI: Montreal Neurological Institute
FWHM: Full width half-,maximum
SPM8: Statistical parametric mapping 8
GLM-Flex: Set of second level neuroimaging analysis scripts.
